# Biotechnological Tools to Elucidate the Mechanism of Plant and Nematode Interactions

**DOI:** 10.3390/plants12122387

**Published:** 2023-06-20

**Authors:** Arshad Khan, Shaohua Chen, Saba Fatima, Lukman Ahamad, Mansoor Ahmad Siddiqui

**Affiliations:** 1 Department of Botany, Aligarh Muslim University, Aligarh 202002, India; fatimasaba8272@gmail.com (S.F.); lukmanamu@gmail.com (L.A.); mansoor_bot@yahoo.com (M.A.S.); 2 National Key Laboratory of Green Pesticide, Guangdong Province Key Laboratory of Microbial Signals and Disease Control, Integrative Microbiology Research Centre, South China Agricultural University, Guangzhou 510642, China; 3 Guangdong Laboratory for Lingnan Modern Agriculture, South China Agricultural University, Guangzhou 510642, China

**Keywords:** root-knot nematode, effector, transcriptomics, metabolomics, CRISPR/Cas-9, QTL

## Abstract

Plant-parasitic nematodes (PPNs) pose a threat to global food security in both the developed and developing worlds. PPNs cause crop losses worth a total of more than USD 150 billion worldwide. The sedentary root-knot nematodes (RKNs) also cause severe damage to various agricultural crops and establish compatible relationships with a broad range of host plants. This review aims to provide a broad overview of the strategies used to identify the morpho-physiological and molecular events that occur during RKN parasitism. It describes the most current developments in the transcriptomic, proteomic, and metabolomic strategies of nematodes, which are important for understanding compatible interactions of plants and nematodes, and several strategies for enhancing plant resistance against RKNs. We will highlight recent rapid advances in molecular strategies, such as gene–silencing technologies, RNA interference (RNAi), and small interfering RNA (siRNA) effector proteins, that are leading to considerable progress in understanding the mechanism of plant–nematode interactions. We also take into account genetic engineering strategies, such as targeted genome editing techniques, the clustered regularly interspaced short palindromic repeats (CRISPR)/CRISPR associated protein 9 (Cas9) (CRISPR/Cas-9) system, and quantitative trait loci (QTL), to enhance the resistance of plants against nematodes.

## 1. Introduction

In the upcoming years, ensuring food security and feeding the growing world population will be major challenges. Food accessibility from a physical, social, and economic perspective, as well as food safety, sufficiency, and nutritional properties, continue to be important in the current period [1]. Nematodes are animals that belong to the phylum Nematoda. In existence for almost a billion years, these are multicellular animals [2]. Several nematodes are parasites of plants and animals, but others may live independently [3].

Plant-parasitic nematodes (PPNs) are tiny roundworms that live in a range of habitats. PPNs are a persistent threat to sustainable agriculture, causing crop losses worth an estimated USD 100 billion each year [4,5]. To date, over 4100 plant-parasitic nematode (PPN) species have been reported [6]. Among all, the most common and devastating PPN is the root-knot nematode (*Meloidogyne* spp.; RKN) [7,8]. *Meloidogyne* is an obligate, sedentary endoparasite found worldwide and can potentially afflict almost any vascular plant, whether in protected agriculture, greenhouses, or the field [9]. There are more than 100 species in this genus, but four of them are responsible for the majority of crop production losses: the tropical species *M. incognita*, *M. arenaria*, and *M. javanica*, as well as the temperate species *M. hapla* [7]. The root-knot nematodes (RKNs) have a diverse host range and affect a wide variety of agricultural crops and wild plants [10,11]. Over 3000 plant species have been identified as being attacked by *Meloidogyne* species, resulting in yearly losses in the billions of dollars [12,13]. In this review, we summarize the various strategies for implementing the knowledge about the biology of the RKNs, molecular plant–nematode interactions, molecular strategies, molecular genetics strategies, proteomics, metabolomic strategies, and genome engineering strategies towards reinforcing nematode control.

## 2. Biology of Root-Knot Nematodes

Plant-parasitic nematodes (PPNs) are microscopic, diverse groups of animals. They are predominantly free-living, but some are parasitic [14]. Root-knot nematodes (RKNs) are found throughout the world. According to the scientific community, *Meloidogyne* is considered one of the top ten PPNs [7]. They are worm-like, smooth, un-segmented, multicellular, pseudocoelomate, and bilaterally symmetrical. Females become obese at maturity and have spheroid and pear-shaped bodies [15]. RKNs are obligate biotrophic, endoparasite-induced giant cells that obtain their nutrients from the root vascular tissues. Numerous studies cover all aspects of *Meloidogyne’s* existence, including its evaluation, development, and virulence, as well as plant responses to nematode invasion [3,16]. Sikandar et al. [17] stated that about 100 species of RKNs are in the genus *Meloidogyne*, which shows how important it is for the economy. A recently published work demonstrated that the parasitism regulatory landscape of *Meloidogyne* was secured at developmental stages by transcriptome analysis [18]. Castagnone-Sereno et al. [19] suggested that *Meloidogyne* is well-adapted to fluctuating environmental conditions and, therefore, is a good model for the study of plant–nematode interactions. Numerous *Meloidogyne* species reproduce asexually by meiotic depletion and subsequent reestablishment of chromosomal numbers through the fusion of the second polar nuclei along the egg pronuclei [20]. Asexually reproducing species include *M. hapla*, *M. exigua*, *M. chitwoodi*, and *M. fallax*. Parthenogenesis is followed by some species of *Meloidogyne*, including *M. javanica* and *M. incognita* [20]. Baniya et al. [21] conducted research and concluded that asexual reproduction may generate males epigenetically, which is feasible under environmental stress scenarios; however, the sperm nucleus is damaged upon female insemination. Some minor species of *Meloidogyne* (*M. megatyla*, *M. microtyla*, *M. pini*, and *M. carolinensis*) prefer to reproduce sexually [22].

### 2.1. Life Cycle of Root-Knot Nematodes

**Figure 1:**
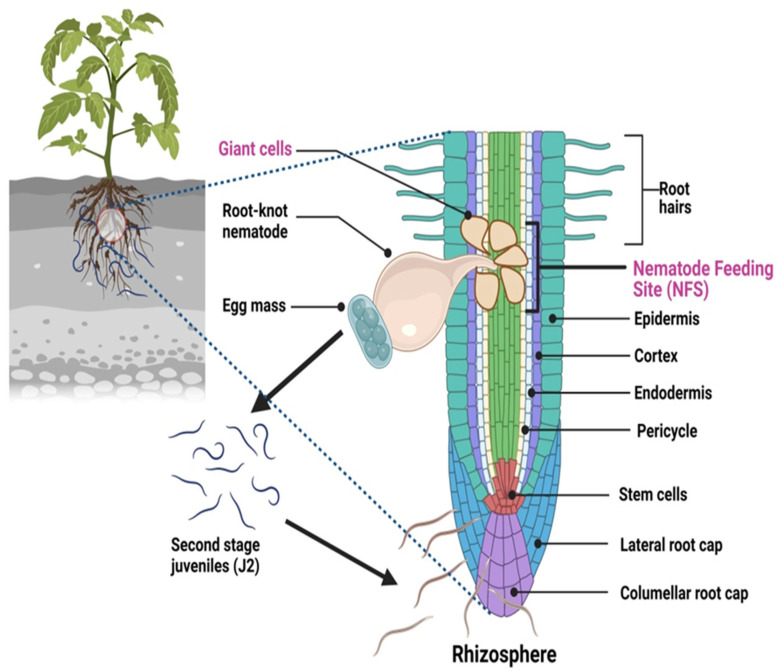
Life cycle of root-knot nematode in the rhizosphere.

Root-knot nematodes (RKNs) can complete their life cycle within 20–40 days at 27 °C, but the duration of their life stages are influenced by environmental factors, including temperature, soil moisture and, to a lesser extent, host species [23]. Approximately 500 eggs are laid by the female into a viscous matrix secreted by 6 anal glands [9]. A gelatinous mass, which consists of a glycoprotein matrix, helps to protect eggs from environmental extremes, serves as a humidity sensor for developmental progress, and keeps the eggs together [9,24]. The eggmasses are usually located on the surface of a gall-infected root and are found embedded within the galled tissue. Vieira et al. [25] found that the vulval secretions of RKNs include a carbohydrate-binding domain (CBD). Six stages (the egg stage, four juvenile or larval stages, and the adult stage) are found in the transition from egg to adult, which takes 25–30 days. Embryogenesis begins inside the egg and develops first-stage juveniles (J1s), which then molt into second-stage juveniles (J2s), which are infective. A glycosphingolipid (a branched long-chain sphingoid base that is rarely seen in nature) was detected in the eggs and juveniles of the RKN *M. incognita* [26,27]. The hatching of J2 nematodes is primarily dependent on temperature, moisture, and other factors, such as generation, root diffusate, and hatching response modification, which provide suitable conditions for the movement of the J2s and a suitable host location [28]. The hatched J2s undergo a period of developmental arrest. This process is somewhat functionally similar to the clearly described genetic pathway in *Caenorhabditis elegans*, a free-living nematode [27], which results in the formation of a developmentally arrested stage termed dauer that is controlled by Dougherty [29]. The dauer pathway genes encode a complex web of signal transduction proteins, which help to detect food, other environmental signals, and pheromones and proceed at the cellular level to promote metabolic activities and control reproductive development and lifespan [30]. The nematode juveniles (J2s) in the soil are vulnerable and require a suitable host to complete their life cycle. The invasive J2s penetrate inside the root tissue with the help of their piercing tool, commonly known as a stylet, and start feeding. The J2s develop a permanent feeding relationship with the protoxylem and protophloem cells, stimulating these tissues to differentiate into a special kind of nurse cells called giant cells (Figure 1).

Several investigations into the gene expression of giant cells demonstrated that the mRNA for a few genes could be many times more abundant in giant cells than in non-infected root cells [31,32]. After the formation of giant cells, the nematode becomes sedentary and gains a sausage-like appearance. Favorable conditions push the J2s to molt into third-stage juveniles (J3s), and another molt gives rise to fourth-stage juveniles (J4s). J3s and J4s have a non-functional stylet, and the J4 stage shows sexual dimorphism, thus distinguishing male and female nematodes [9]. If males are found, they appear vermiform, emerge from the root, and become free to live in the soil, whereas the sedentary mature female resumes feeding and appears to have a sausage or pear shape. She continues to grow and produce eggs. Different gene expressions encompass these transition processes; for example, sensory perception and cell wall degeneration genes are upregulated during the transition from eggs to the pre-parasitic mobile phase (J2), whereas stress response genes are upregulated during the transition from the J2 to J3 or J3 to J4 phases and sensory genes regarding perception are downregulated. In preparation for the active adult stage, lipid-metabolism-related genes are upregulated during the J3 and J4 phases, but mature females inhibit these genes due to their lack of locomotion [9]. The control of egg-related genes, which include genes related to membrane transport and DNA metabolism, is mostly determined by the stage of development. Iqbal et al. [33] demonstrated that the pattern of the gene expression of RKNs provides a strategy for the reasonable choice of target genes; however, different findings have been observed, and the RNA silencing of only a small set of genes has been shown to reduce the infestation level.

### 2.2. The Genomes of the Root-Knot Nematodes

Genomics information provides a burgeoning fundamental base that, combined with downstream functional genomics and proteomics, can accelerate the progress of sustainable control programs and foster an interest in the study of plant and nematode interaction. The tomato–root-knot nematode (RKN) system is a great crop model for understanding how a host reacts to a pathogen because both tomato and RKN have relatively well-annotated genome reference sequences [34]. An expressed sequence tag (EST) analysis of infective *M. incognita* J2s identified many cell wall hydrolytic enzymes as the first genomic approach [35]. Several putative effectors were identified in the first *M. incognita* genome draft that was released in 2008 [36]. Whole-genome resequencing, genetic analysis, genome-wide association investigations and haplotype assessments have been used to map and examine genomic sites for nematode resistance [37]. Sequencing analysis revealed the current completion of two RKN genomes, which provides a pathway for a comparative genomics approach to understanding the success of parasites. The 54 Mbp genome of the diploid *M. hapla*, which reproduces through facultative, meiotic parthenogenesis, has around 14,200 genes. Undoubtedly, this is the smallest metazoan genome yet sequenced. The 86 Mbp genome of *M. incognita*, on the other hand, encodes nearly 19,200 genes. This species reproduces by mitotic parthenogenesis and has a complex aneuploidy pattern [38].

Numerous different nematode genomes, such as those of *Caenorhabditis*, free-living nematodes, and nematode parasites of humans and animals, have been sequenced to different degrees of coverage (http://www.ncbi.nlm.nih.gov/projects/genome/guide/nematode/index.html, accessed on 15 December 2022). There is much evidence that whole-genome duplication (in *M. incognita*) and horizontal gene transfers (HGTs) are the most important processes that have changed the genomes of living RKN species. Perhaps this is the reason for nematodes’ ability to spread and adapt to a broad range of environments. Asexually reproducing species have more duplicated sites due to the abundance of transposable elements (TEs), suggesting the functional delineation of the duplicated sites and genome plasticity [39]. Phan et al. [40] identified 575 canonical TEs from seven orders, accounting for 2.61% of the genome. These TEs are believed to promote genomic plasticity as a result of the progression of parasitism in *M. graminicola*. This high-quality genome assembly represents a significant improvement over previous versions and a precious molecular resource for future *Meloidogyne* phylogenomic research. Phan et al. [40] explored the production of extremely consecutive genomic sequences using a combination of Oxford Nanopore Technologies and Illumina sequence information (283 scaffolds with an N50 length of 294 kb, totaling 41.5 Mb). Susic et al. [41] published the genome sequence of the *M. luci* population SI-Smartno V13. The SI-Smartno V13 genome assembly consists of 327 contigs with a total assembly length of 209.16 Mb and an N50 contig length of 1,711,905 bp. Koutsovoulos et al. [42] used both short-read and long-read methods to sequence the genome of *M. enterolobii*. High-quality annotations of 59,773 coding genes, 4068 non-coding genes, and 10,944 transposable elements (covering 8.7% of the genome) are made possible by the 240 Mbp genome assemblies with a contig N50 size of 143 kbp. Many important agronomic genomes of RKN species were sequenced using short-read sequencers [36,39,43]. The genomes of asexual *Meloidogyne* are polyploid and encompass duplicated areas with high nucleotide divergence (8%) [36,44]. In essence, asexual *Meloidogyne* genome assemblies, including *M. javanica*, *M. incognita*, and *M. arenaria*, are highly fragmented when compared to the *M. hapla* genome assembly [39,45]. Blanc-Mathieu et al. [39] sequenced the genomes of three asexually reproducing RKN species, with the assemblies for *M. arenaria*, *M. javanica*, and *M. incognita* reaching 258, 236, and 184 Mb, respectively (Table 1). These genome assemblies are bigger than any RKN genome assembly previously reported.

### 2.3. Exploration of Available Resources for Research of Root-Knot Nematodes

The computational analysis of genes and proteins and their roles in parasitism is quickly becoming one of the most important methods for studying plant–nematode interactions. Many computational tools and databases are freely available for the use of researchers to understand the interactions between plants and nematodes. WormBase, a sophisticated tool for nematode research, was developed for *C. elegans* [47]. It includes information about parasitic nematodes as well as more recent *Meloidogyne* sequences (https://parasite.wormbase.org/index.html, accessed on 21 January 2023). WormBase contains all of the information currently available on the genes and genomes of the 138 distinct nematode species. *Meloidogyne* genomic resource (https://meloidogyne.inra.fr, accessed on 29 January 2023) is a database maintained by the INRA that provides data on three root-knot nematode (RKN) species: *M. javanica*, *M. incognita* and *M. arenaria*. An additional RKN database available at http://nematode.net/NN3 frontpage.cgi is also quite useful [48]. Some other automated systems have been characterized with the goal of tracking single or multiple worms and studying their movement and behavior quantitatively [49,50]. For example, Nemo is an algorithm that uses video image sequences to measure and analyze nematode movement characteristics [51]. In 2008, the 86 Mb and 54 Mb genomes of *M. incognita* and *M. hapla*, respectively, were sequenced [36,45]. Genomic and phylogenetic comparisons are made possible by the discovery of 19 more genome drafts for 6 species at (https://www.ebi.ac.uk/ena/browser/view/PRJNA340324, accessed on 29 January 2023) [2,43]. This database includes data about the functional genomes, transcriptomics, and proteomics of all parasitic nematodes.

## 3. Molecular Strategies

In addition to rapid structural changes in the cell morphology of nematode-infected roots, gene expression at the whole plant level is seriously influenced. A variety of approaches have been used to investigate these transcriptional changes. Microarrays are associated with approaches for isolating RNA from specific plant tissues, such as laser microaspiration and microdissection. These approaches allow for a comprehensive microanalysis of the transcriptional alterations prevailing in the syncytia and giant cells during the initial phases of differentiation. The development of technology has facilitated genomic, transcriptomic, metabolomic, and proteomic investigations of plant and nematode interactions.

### 3.1. Transcriptomic Technology

Undoubtedly, transcriptomic analysis has significantly contributed to the understanding of the molecular mechanisms of plant-parasitic nematodes (PPNs) in parasitism [52]. The first line of defense is pathogen recognition by the plant, which stimulates signaling pathways that trigger the transcription of plant defense genes [53]. Researchers were able to compare the transcriptome data of four root-knot nematode (RKN)-resistant plant species (*Glycine max*, *Arachis stenosperma*, *Oryza glaberrima*, and *Coffea arabica*) infected with three different *Meloidogyne* spp. (*M. incognita*, *M. graminicola*, and *M. arenaria*) using a database called a homology database [54,55,56]. Transcriptome analysis is an effective tool for estimating transcriptional changes in cells under various conditions [57,58]. Transcriptomic studies assist in the identification of individual resistance genes involved in each of these steps of the defense system by providing knowledge about the genes and metabolic pathways that are differentially regulated during plant–pathogen interactions [59,60]. Whole-transcriptome RNA sequencing (RNA-seq) is a promising method for studying differential gene expression in plant–pathogen interactions [61]. Information on defense genes can be valuable for marker-assisted selection (MAS) as well as for choosing genes whose altered expression through transformation can result in enhanced resistance.

Over the last decade, multifaceted molecular biology techniques, such as microarrays, RNA blotting, EST sequencing, differential cDNA library screening, and more recently, next-generation sequencing (NGS), have all been used to investigate the transcriptomic changes that take place in either susceptible or resistant plants during host and RKN interactions [62]. Microarray-based transcriptomic investigations were carried out in tomato [63,64], Arabidopsis [65,66,67], and RKN-tolerant aubergine, *Solanum torvum* [68]. Microarray technology facilitates the generation of large-scale data on gene expression patterns during plant–nematode interactions [69]. Understanding the complexity of plant–nematode interactions through the investigation of transcript abundance changes throughout feeding site establishment may encourage the development of innovative nematode management techniques. *M. javanica* altered the transcriptome of large cells in *Arabidopsis* relative to root vascular cells three days after infection. Laser microdissection was used to collect giant cells and root vascular cells for a microarray study that was confirmed by qPCR and a promoter-GUS fusion assay. Although some genes were regulated similarly in galls and GCs, the majority had distinct expression patterns, elucidating the molecular demarcation of the GCs within the gall [67]. Previous research has suggested that plant–nematode interactions influence the expression of genes involved in plant immune reactions in dicotyledonous plants [67,70]. Through RNA-seq transcriptome analysis, Zhou et al. [71] explored the invasion and development of RKN in rice roots. At 6 (the invasion stage) and 18 (the development stage) days after inoculation, 952 and 647 genes were differentially expressed. Moreover, 40 new differentially expressed genes of RKN encoding secretory proteins were observed [34].

### 3.2. Effector Molecules

In a broad sense, effectors, defined as nematode-derived molecules (often proteins) secreted into the host, have developed to manipulate different facets of host metabolism, physiology, development, and immunity in order to make a host susceptible [72]. The plant-parasitic nematodes (PPNs) produce effector proteins to control the molecular and physiological pathways of their host for their own advantage. The formation of unique, highly specialized feeding cells in the roots of the host plant is a key step in the process of infection that leads to a successful parasitic relationship. Root-knot nematodes (RKNs) are obligatory biotrophic parasites that infest plant roots and establish stable, long-term relationships with their hosts. Although nematode secretions, some of which have immunosuppressive activity, play critical roles in parasitism, their exact mechanisms of action are largely a mystery [73]. In the various parasitic phases of the nematode, both the synthesis and secretion of effector proteins are developmentally regulated in the esophageal glands [74,75]. Similar to other plant pathogens, RKNs produce effector proteins in plant cells in order to change host cell processes and facilitate parasitism. The esophageal gland, hypodermis, amphids, and phasmids are all nematode organs that can secrete effectors [76]. Nematode esophageal gland secretions are thought to encourage the development of root cells close to the vascular system into intricate feeding sites [77]. The effector proteins involved in parasitism, whether directly or indirectly, are referred to as “secretomes” or “parasitomes”. The parasitomes of sedentary endoparasitic nematodes, particularly those belonging to the genera *Meloidogyne*, *Globodera*, and *Heterodera*, are particularly intriguing because they induce dramatic gene expression patterns in parasitized plant cells, resulting in intricate morphological, biochemical, and metabolic changes that transform parasitized root cells into distinctive nematode feeding sites (NFSs) [78].

**Figure 2:**
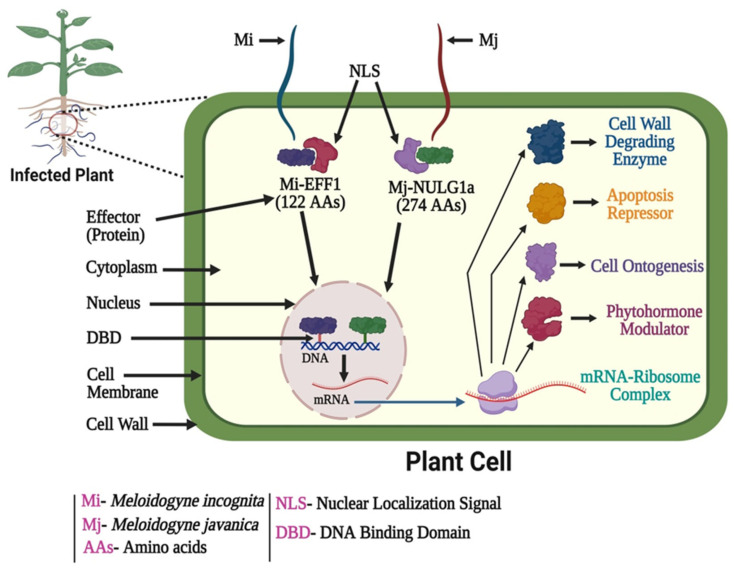
Schematic model of the interactions of root-knot nematodes (*M. incognita* and *M. javanica*) with host plant cell.

Genes encode for enzymes that degrade or modify plant cell walls enzymes, such as chitinases [79], pectinases [80,81], cellulases or -1,4-endoglucanases [79,82], and xylanases [45]. These cell-wall-degrading enzymes facilitate the migration of infective J2 nematodes through plant roots by smoothing the cell wall. After penetration, recent research has demonstrated that RKNs deliver two effectors into the cytoplasm of giant cells; these proteins target the nucleus [83]. Jaouannet et al. [84] conducted immunolocalization assays on infected tomato roots and validated the secretion *in-planta* of Mi-EFF1, a peptide (composed of 122 amino acids) with a nuclear localization signal (NLS). The secreted Mi-EFF1 was found in the nucleus of giant cells. It appears that Mi-EFF1 is only involved in the initial stages of the interaction between plants and RKN. Likewise, the Mj-NULG1a protein (274 amino acids) of *M. javanica* consists of a signal peptide for secretion, with two NLS domains. The dorsal gland of the RKN is responsible for the production of this effector, which is then delivered into the cytoplasm of the giant cells. Immunocytochemical assays of infected tomato roots have shown that it then builds up in the nuclei [85]. The cell cycle and transcriptional regulation are only two examples of host nuclear activities that must be regulated during giant cell ontogenesis and maintenance by the delivery of secreted effectors. Various previous studies suggested that RKN effectors target different nuclear processes to change plant cellular proliferation and immunity in ways comparable to those seen with other phytopathogens [86,87]. These examples show how RKNs manipulate host plant roots using a variety of effectors. To summarize this process, we propose a schematic model (Figure 2).

MiMsp40 was isolated as a clone from an *M. incognita* gland-specific cDNA library approximately a decade ago; nevertheless, little is known about its roles in nematode–plant interactions. Niu et al. [73] found that in Arabidopsis, the constitutive expression of MiMsp40 resulted in morphological changes and increased susceptibility to *M. incognita*. Furthermore, the effector MiPFN3 of *M. incognita* stimulates the creation of feeding sites by directly remodeling the actin proteins of plant cells, resulting in the development of giant cells [88]. MiSGCR1, a tiny cysteine- and glycine-rich effector, has been identified as playing a vital role in parasitism by inhibiting hypersensitive-reaction-induced plant cell death [89]. Another RKN effector, MiEFF18, interferes with SmD1, a plant core spliceosomal protein that is required for the formation of giant cells [90]. Three rice defense-related proteins (OsGSC, OsCRRSP55 and OsBetvI) have been shown to interact with the *M. graminicola* effector MgMO237 [91]. In searching for effector-like proteins, many software programs are used. PSCSORT (http://psort1.hgc.jp/form.html, accessed on 16 February 2023) and PHOBIUS (http://phobius.sbc.su.se/, accessed on 19 February 2023) software (1.01 standalone) have been used to assess signal peptides, non-trans-membrane domains, DNA-binding domains (DBD) and nuclear localization signals (NLS) from the protein sequences encoded by the genes [89]. Regarding candidate proteins, NTERPROSCAN was used to find protein annotations in the InterPro database [92]. Various nematode effectors are functionally elucidated in Table 2. These effectors are involved in both compatible and incompatible interactions between plants and nematodes. However, many remained unclear because most effectors have yet to be identified.

## 4. Molecular Genetics Approaches

Scientists have been searching for various innovative tools since the beginning of the century. Emerging biotechnological tools have given a lot of impetus to agricultural research, which has greatly improved our understanding of host–pathogen interactions. The emergence of novel molecular and genetic approaches that facilitate the detailed investigation of gene expression and regulation in giant cells, as well as these technologies applied to both the host and the nematode, is leading to rapid developments in the understanding of host–nematode interactions. Therefore, in order to develop stable resistance and maintain agricultural productivity, it is essential to understand the molecular and genetic pathways that confer protection to plants against root-knot nematodes [109]. To impose molecular functions, alternative techniques such as the siRNA and RNAi approach, quantitative trait loci (QTLs), yeast 2-hybrid, in situ hybridization and pull-down experiments are being used [72].

### 4.1. siRNA Technology

**Figure 3:**
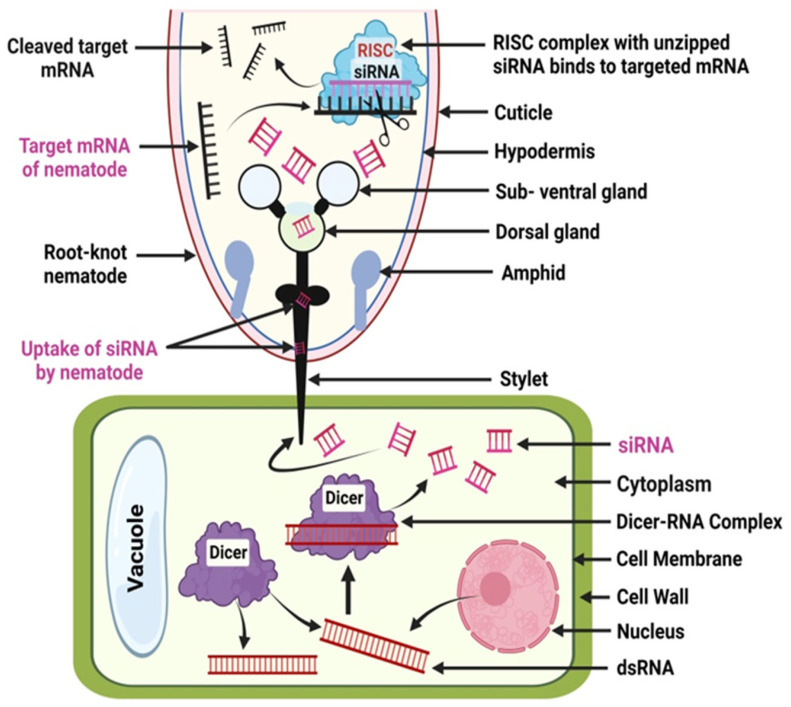
Host plant cell and root-knot nematode interaction-based siRNA production.

Host-generated small interfering RNA (siRNA) has proven to be an innovative way to transfer dsRNAs into feeding nematodes to silence vital genes found in nematodes. Those genes whose expression is necessary for the nematodes to start feeding should be purposefully targeted. The expression of the dsRNA might be driven by a tissue-specific or constitutive promoter. The dsRNAs can be processed by the RNAi machinery of host plants, resulting in siRNAs ingested by the plant-parasitic nematodes [11,110] (Figure 3). Recently, researchers have made significant progress in understanding how to use siRNA technology to suppress the expression of these nematode effector genes, therefore diminishing nematode parasitism. The fundamental concept is to introduce an expression cassette into host plants that generate dsRNAs that target one or more genes vital for nematode infection. It requires intricate molecular mechanisms to produce siRNAs from dsRNAs or hairpins. The type III RNAse enzyme Dicer converts double-stranded RNAs into siRNA duplexes, typically 19–26 bp in length, with a 2-nucleotide 3′overhang [111,112]. During host infection, the nematode ingests dsRNA-derived siRNAs via its stylet, causing the inhibition of target gene expression and hence impeding effective nematode parasitism [113]. The nematode RNAi machinery then unzips the siRNAs from the host and binds them to the RISC complex, where they cleave the target mRNA sequence-specifically, preventing the mRNA from being translated (Figure 3). Such a method has been proven with success in transgenic tobacco [114], Arabidopsis [115,116], and soybean [117] for controlling root-knot nematodes.

### 4.2. RNAi Technology

The finding of RNA interference (RNAi) in the free-living nematode *C. elegans*, in which double-stranded RNA (dsRNA) causes endogenous genes with similar sequences to be turned off after transcription, has given scientists an entirely novel approach to studying how genes interact [118]. Therefore, RNA interference can be utilized to develop transgenic plants that use RNAi to reduce the risk of plant-parasitic nematodes (PPNs). RNAi has recently emerged as one of the most promising approaches to managing PPNs. The most suitable strategy for developing nematode resistance in plants is the host-mediated RNAi approach, which targets nematode genes and involves both plant and nematode RNAi machinery [119]. To facilitate successful infections, root-knot nematodes (RKNs) secrete proteins called effectors that hijack host responses. As a consequence, RKN effector genes are targets for RNA interference gene silencing (RNA interference). By rinsing nematodes with dsRNA, the targeted nematode gene can be silenced. Parasitic success is negatively affected, while nematode effector genes that are required for parasitism are silenced. The cyst nematodes *Heterodera glycines* and *Globodera pallida* were the first to demonstrate RNAi of PPN genes [120]. Rosso et al. [121] used RNAi for the first time on *M. incognita* J2s to turn off two genes, Mi-pg1 and Mi-crt, that are active in the sub-ventral esophageal glands of nematodes and seem to play a role in the early stages of infection. This was followed by host-induced gene silencing (HIGS) on *M. incognita* and *H. glycines*, a common technique in which transgenic plants are engineered to produce long hairpin RNAs targeting essential nematode genes. These RNAs are then converted into short interfering RNAs (siRNAs) that cause gene silencing when absorbed by nematodes [115,122].

Transgenic plants that are resistant to nematodes have also been developed by targeting multiple genes. The two housekeeping genes of *M. incognita* splicing factor and integrase were targeted for nematode resistance engineering using a host-delivered RNAi (HD-RNAi) approach [123]. HD-RNAi of the putative effector gene Mc16D10L leads to significant resistance to *M. chitwoodi* in potatoes and *Arabidopsis* [124]. Genetically modified crops that express dsRNAs, specifically in roots, to interrupt the parasitic process provide an efficient and effective method of producing resistant crops [125]. In most RNAi-based nematode control strategies, the constitutive promoter CaMV35S is used to generate dsRNA in the host plant [126]. Mi-msp-1 silencing also hampered the development and reproduction of *M. incognita* in adzuki beans [127]. Chan et al. [128] developed a synthetic promoter termed pMSPOA with NOS-like and SP8a elements to confer PjCHI-1 and CeCPI genes to transgenic plants of tomato. These double-gene transgenic plants of tomato significantly decreased RKN infestation and reproduction compared to those converted with a single gene. When the parasitism gene 16D10 was utilized for RNAi in the model plant *Arabidopsis thaliana*, the gall number and fecundity of *M. incognita* were drastically reduced (63–90%) [115]. Therefore, RNAi technology can be a potent and highly targeted technique for combating nematode parasitism genes.

### 4.3. Quantitative Trait Loci (QTLs)

Plant molecular genetics has been used to find quantitative trait loci (QTLs) or nematode resistance (R) genes for resistance against sedentary endoparasitic nematodes. Several genes have been mapped to linkage maps or chromosomal locations. There are two major challenges to QTL mapping in biparental populations: (1) it can only investigate the allelic variations between the populations of two parents; (2) few recombination events are normally acquired in these kinds of populations, restricting genetic resolution [129]. The majority of strategies are based on the temporal and spatial management of R-gene implementation: (1) the modification of different R-genes in crop rotation, (2) the use of combinations of varieties with different R-genes, or (3) pyramiding, which is the emergence of multiple R-genes into the same variety [130,131]. There are numerous QTLs that provide resistance to soybean cyst nematode (SCN), but only two are commonly used: rhg1 and Rhg4 [132]. Earlier, QTLs controlling nematode resistance were discovered on *Arachis stenosperma* chromosomes A02, A04, and A09 [133]. QTL introgression into improved varieties, in particular, may not hamper other agriculturally important crop characteristics, such as production, quality standards, adaptation, or other physiological properties. QTL analysis was used to analyze the genetic perspectives that were shown earlier to influence the efficacy and durability of resistance. Biparental populations have mainly been used to identify QTL associated with resistance to *M. incognita* [134,135]. The genes for resistance to the southern RKN (*M. incognita*) were identified using quantitative trait loci (QTL) analysis. QMi-LG2, a major QTL with a LOD of 14, explains 32.0% of the phenotypic variance. This nematode-resistance QTL was discovered in a distal region of pearl millet LG2 [136]. Zwart et al. [137] reported that the two QTLs resistant to wheat root-lesion nematodes (*Pratylenchyus thornei* and *P. neglectus*) were observed on the short arm of wheat chromosome 6D. Mapping and identifying QTLs responsible for *M. graminicola*’s resistance and tolerance may provide growers with a safe and cost-effective management option [138].

### 4.4. Genetic Engineering Strategies

Genetic engineering presently provides a new breeding opportunity, which is the direct insertion of a previously isolated gene into the genome of the desired organism. The most preferable, cost-effective, and environmentally friendly method for managing plant-parasitic nematodes (PPNs) is the innovation of resistant cultivars that restrict nematode growth and proliferation. Host plant resistance is only effective against particular species or races of root-knot nematode (RKN); therefore, proper classification of RKN species is critical to the success of host resistance [139]. Several genes conferring resistance to a variety of plant pathogens have been successfully transferred in tomato, and the transgenic plants showed reduced disease incidence [140].

#### CRISPR/Cas9 Technology

**Figure 4:**
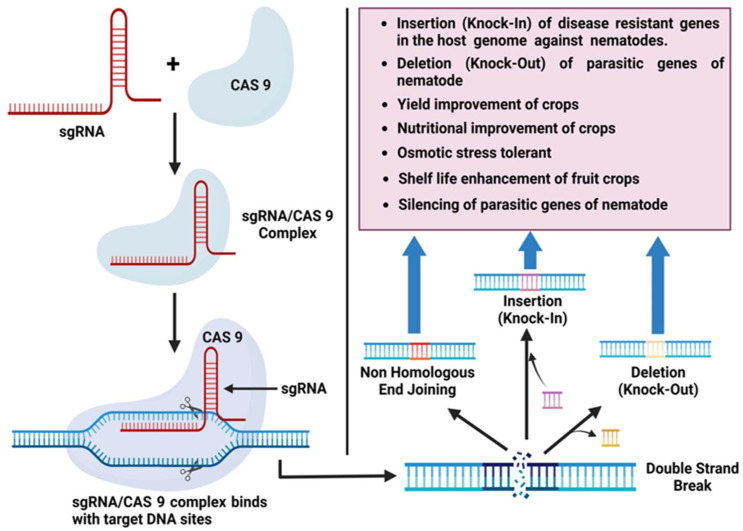
Overview of CRISPR/Cas9 technology to target multiple genes to develop resistance against root-knot nematodes.

The recent technological advancement of genetic engineering has revolutionized both fundamental and applied plant breeding research. The current scenario allowed for the efficient explorations of molecular insight by targeting different genes to produce resistant plants. In the 1990s, Ishino et al. [141] discovered clustered regularly interspaced short palindrome repeats (CRISPR) and performed the first characterization of the CRISPR-Cas system. The term CRISPR was later coined by Jansen et al. [142]. CRISPR/Cas9 has recently emerged as the most effective and simple genome editing tool at the genomic level [143]. It is a burgeoning genome editing strategy that is being used effectively in diverse organisms, including model and crop plants. Several cereal crops, including rice, wheat, maize, and barley, have successfully undergone genome editing using the CRISPR/Cas9 system [144]. The availability of whole-genome sequence information for a range of crops, combined with advances in genome-editing tools, opens new opportunities for attaining desirable traits [144]. In the CRISPR/Cas9 system, tracrRNA and crRNA are joined to generate a single guide RNA chimera (sgRNA) that also coordinates the sequence-dependent dsDNA break induced by Cas9. The Cas9-sgRNA complex attaches to the target site where the sgRNA matches the homologous sequence, resulting in a double-strand break (DSB) [145]. Genome editing is a rapidly expanding field. The editing of nucleases has revolutionized genomic engineering by permitting simple genome editing. Engineered nucleases are classified into four types: (1) zinc-finger nucleases, (2) meganucleases, (3) transcription-activator-like effector nucleases (TALENs), and (4) clustered regularly interspaced short palindromic repeats (CRISPR) systems. Later, several different but closely related techniques that had all been improved for use in genome editing were put together [146]. Most applications now use either CRISPR systems or TALENs. It has been revealed that CRISPR/Cas9-directed genome editing in plants is effective in chickpeas, the legume model *M. truncatula*, and *G. max* [99,147]. Recently, this approach was utilized to develop many characteristics in corn by grouping the transgenes into a single complex trait locus [148]. This provides intriguing opportunities for gene pyramiding and trait stacking in several plants, including soybean, to attain a consistent degree of resistance. To resolve some of the obstacles involved with this technology, a CRISPR-Cas9 genome editing platform in soybean was developed, which should assist future studies [149]. CRISPR/Cas9 gene-edited plants are recognized as genetically modified, hence representing a powerful tool in combating root-knot nematodes (Figure 4).

## 5. Physiological Approaches

Root-knot nematode (RKNs) infection alters host metabolism by interfering with the physiological and biochemical pathways of host plants [150]. They modify the physiological and transport mechanisms of vascular tissues inside the host plant, altering the synthesis and flow of primary and secondary metabolites and transforming feeding areas into nutrient sinks and immune suppression sites. In particular, there are more amino acids and sugars at NFSs, and alterations also occur in the overall metabolism of vital amino acids and carbohydrates [151].

### 5.1. Metabolomics

Supporting breeding programs with a metabolomics method is feasible. This method allows plant breeders to assess chemicals that play a crucial role in plant resistance to pests and diseases. Plants that are resistant to pests and diseases produce a wide variety of biologically active chemical compounds and secondary metabolites. Chromatographic techniques, such as gas chromatography (GC), liquid chromatography (LC), thin layer chromatography, capillary electrophoresis, nuclear magnetic resonance (NMR) spectroscopy, and mass spectrometry (MS), are commonly used in metabolomics [152,153,154,155]. Martnez-Medina et al. [156] investigated the expression profile of a group of oxylipin-linked marker genes and the jasmonate content of the root. They found that the *M. incognita*-induced inhibition of the 13-LOX branch is counteracted by leaf herbivory systemic activation of the 9-LOX branch of the oxylipin pathway in the roots. Natural plant compounds exhibit several biological activities that may be related to resistance to a variety of pests and pathogens, including RKNs [157,158]. Nematotoxic (nematicidal and/or nematostatic) effects in plants are predicated on compounds such as isothiocyanates, thiophenes, glucosides, alkaloids, phenolics, tannins, and others that have been recognized [159].

### 5.2. Phytoalexins and Phytoanticipins

The interplay of second messengers with the infected cell’s genome results in the accumulation of compounds, the majority of which are deleterious to pathogens. A current method of classification divides chemicals used by plants as a method of disease defense into phytoanticipins and phytoalexins. Phytoanticipins and phytoalexins are two categories of biocidal secondary metabolites produced by plants as a defense against pathogens and pests. The term “phytoanticipins” refers to defense compounds that are always present, regardless of the presence of pathogens or pests [160]. Contrarily, phytoalexins only build up when pests or pathogens are detected [160]. The first report of phytoalexin production in nematode-infected plants has been published [161]. Plant roots exude defensive compounds in response to an attack (stimulated defense; phytoalexins) or constitutively (phytoanticipins) [160]. These molecules serve as the first line of defense against potential pathogens [162]. The phytoalexin glyceollin, a consequence of the isoflavonoid branch of the phenylpropanoid pathway, is aggregated in the roots of *M. incognita*-resistant soybean plants [163]. In some interactions, plant genotypes with high nematode resistance accumulate elevated levels of flavonoids, which may act as phytoalexins. When the plant-parasitic nematodes (PPNs) reach a plant, they cause mechanical injury to the root tissues in order to penetrate and/or feed on it. Following this, defense compounds (i.e., phytoalexins and phytoanticipins) are produced and released in response to PPN attacks [164].

### 5.3. Volatile Organic Compounds (VOCs)

Small volatile compounds, or VOCs, can be used in fumigation to produce insecticidal or bacteriostatic effects at specific temperatures and pressures [165]. VOCs are typically less poisonous to humans and animals, so the formation and use of biological control agents have a lot of promise. VOCs can increase the resistance of plants to pathogens by activating hormone-dependent signaling pathways in plants, which act as activation signal molecules for genes related to plant resistance [166]. VOCs are secondary metabolites released by plants that have a variety of functions in nature, including the ability to attract, repel, or even kill microorganisms [167]. Additionally, current findings have shown that many VOCs can be retained in water, where they can be held for an extended period of time and act as nematicides for longer [168,169,170]. Various previous studies suggest that VOCs act as nematicidal compounds. The highest nematicidal impacts on the RKN were caused by the VOCs of the plants *C. nardus* and *D. ambrosioides* [171]. The VOCs released by *Azadirachta indica*, *Brassica juncea*, *Canavalia ensiformis*, *Cajanus cajan*, and *Mucuna pruriens* exhibit nematicidal activity against *M. incognita* [172]. Mei et al. [173] were the first to show that VOCs of *Duddingtonia flagrans* have nematicidal potential against *M. incognita*. de Freitas Silva et al. [171] investigated 15 medicinal plants to determine which species produce the most poisonous VOCs against *M. incognita*.

## 6. Conclusions

Root-knot nematodes (RKNs) are obligate biotrophic plant parasites that cause major losses to various crops around the world, and traditional management strategies are insufficient for eliminating this devastating pest. Therefore, we urgently need to develop many innovative strategies to control this serious threat. Thus, knowledge of plant and nematode interactions could identify the crucial roles of genes and proteins that are involved in infection processes and will help to develop resistance against nematodes in crop plants. This review focuses on the several techniques and strategies utilized to understand the molecular mechanism of plant–nematode interactions, such as RKN biology, computational resources, knowledge of genomes, transcriptomes, metabolomics, and genetic engineering technology, as well as some advanced strategies, such as the role of effector proteins in parasitism, gene silencing (RNAi), and gene knockout techniques (CRISPR/Cas9) to control nematode infections. For example, omics technologies provide a potent toolkit for targeted and effective strategies that could be used to better understand plant–RKN interactions. Advances in computational biology and bioinformatics, along with the analysis of huge-scale omics data, now provide an excellent basis for the identification of biological components and processes involved in parasitism and plant response. The transcriptomic analysis of infected plants has identified various upregulated genes that are involved in the development of NFSs. These genes might be suitable targets for site-specific silencing in the syncytia or giant cells to develop plant resistance. Metabolomics studies will also help to identify cell components and signaling pathways and the role of effector genes in the development of NFSs. The rapid progress of genetic engineering technology over the years has revolutionized the field of molecular plant pathology and enabled gene editing systems at genomic levels. However, current genome-editing and gene knockout technologies are far more accurate than traditional methods and provide a better option for developing resistant crops. In addition, the identification and characterization of QTLs and real potential defense genes in resistant plants against RKN would enable us to understand RKN-mediated resistance. It will also be a useful tool for plant breeders, as RKN resistance may be efficiently integrated into genetically modified varieties. In conclusion, in this review, we demonstrated the significant role of innovative technologies in studying plant–RKN interactions. Understanding innovative strategies and their functions in plant–host interactions is critical for developing counter-strategies against RKN infection in agricultural and horticultural crops to control yield losses and increase production.

## 7. Future Prospects

The present review has focused on how biotechnological techniques, in the broadest sense, could speed up studies of plant–nematode interactions. We have highlighted the technological advances made by several research groups in the biology and interactions of plants and nematodes. To manage this detrimental pathogen, biotechnological tools are required to conduct fundamental research assessing pathogen-host interactions and acquiring a greater understanding of the species’ identity, genetic diversity, and parasitism mechanisms. Genomic comprehension of *Meloidogyne* spp. will provide prospects to identify the extensive occurrence of horizontally transmitted genes encoding for novel effectors, which contribute to successful parasitic interactions with plants and the modulation of plant defense systems. New insights into present and future threats, secured by increased knowledge of plant–nematode interactions, will expand opportunities for developing novel management tools as chemical pesticides become less effective and hazardous to our environment as the demand for food production continuously expands. Combating this economically damaging nematode in agricultural production systems would require stronger collaborative research and the combination of expertise from multidisciplinary areas. There will be further development and widespread adoption of the findings of this study because of the positive effects it has on the economy and the environment.

## Figures and Tables

**Table 1:** Genomic information of *Meloidogyne* species.

Root-Knot Nematode Species	Strain Designation	Number of Predicted Genes	Assembly Size (Mb)	Number of Scaffolds	Protein-Coding Region (Mb)	GC Content (%)	References
*Meloidogyne hapla*	VW9	14,220	53.01	3450	-	27.4	[45]
*M. floridensis*	-	-	96.67	58,696	-	30.0	[43]
*M. incognita*	W1	24,714	121.96	33,735	43.7	30.6	[44]
*M. javanica*	VW4	26,917	150.35	34,394	75.2	30.2	[44]
*M. incognita*	V3	45,351	183.53	12,091	-	29.8	[39]
*M. arenaria*	HarA	30,308	163.75	46,509	82.2	30.3	[44]
*M. enterolobii*	L30	31,051	162.97	46,090	NA	30.2	[44]
*M. graminicola*	IARI	10,196	38.19	4304	-	23.1	[46]

**Table 2:** Nematode effectors and their role in parasitism.

Effector Gene/Protein	Nematode Species	Cellular Localization in Nematode	Cellular Localization in Plant	Function in Parasitism	Ref.
Gr-pel-2	*Globodera rostochiensis*	Subventral esophageal glands	Apoplast	Pectatelyases (cell-wall-degrading and migration)	[93]
Mi-PEL 3/Pectate lyase	*Meloidogyne incognita*	Subventral glands	Apoplast	Protein degradation and cell wall modification	[25]
Bx-crt-1	*Bursaphelenchus xylophilus*	Esophageal gland	-	Calreticulin calcium-binding protein, cell-to-cell trafficking, and differentiation of NF cells.	[94]
Mi-CRT/Calreticulin	*M. incognita*	Subventral esophageal gland cells	Apoplast	Overproduction in plant cells increases plant resistance to RKNs	[95]
Mj-FAR-1/Fatty acid and retinol binding protein	*M. javanica*	Cuticle	Apoplasm	Manipulates the lipid-based signaling	[96]
Mj-eng-3/Beta-1,4-Endoglucanase	*M. javanica*	Subventral glands	Apoplasm	Degrades the cellulose of plant cell walls	[97]
MjTTL5	*M. javanica*	Subventral gland	Plastids	Encodes a transthyretin-like protein that may suppress host defenses	[98]
Rs-CRT	*Radopholus similis*	Esophageal glands, gonads, and intestines of juveniles	-	Essential for reproduction and pathogenicity	[99]
Misp12	*M. incognita*	Dorsal esophageal gland	Cytoplasm	Participates in the maintenance of giant cells during parasitism	[100]
MiMsp40	*M. incognita*	Subventral esophageal gland cells	Cytoplasm and nucleus	Suppresses ETI-associated cell death	[73]
HaEXPB2	*Heterodera avenae*	Subventral esophageal glands	Apoplast	Involvement in successful compatibility Interaction J2s	[101]
MeTCTP	*M. enterolobii*	Dorsal gland	Cytoplasm	Suppresses programmed cell death in host plants	[102]
MgGPP	*M. graminicola*	Subventral esophageal gland cells	Nucleus	Suppresses host defenses and enhances nematode parasitism	[103]
MiSGCR1	*M. incognita*	Dorsal gland	Cytoplasm and nucleus	Suppresses plant cell death	[89]
Mg16820	*M. graminicola*	Subventral glands	Apoplast, cytoplasm, and nucleus	Suppresses both the PTI and ETI responses	[104]
MiISE6	*M. incognita*	Esophageal glands	Nucleus	Suppresses programmed cell death in hosts	[105]
MiPDI1	*M. incognita*	Secreted by the esophageal glands	Cytoplasm and nucleus	Increased susceptibility and facilitates parasitism	[106]
MiEFF1	*M. incognita*	Esophageal glands	Nucleus	Interacts with cytosolic glyceraldehyde-3-phosphate dehydrogenases to promote parasitism	[107]
MiEFF18	*M. incognita*	Salivary glands	Nucleus	Giant cell ontogenesis	[90]
Mi-ISC-1	*M. incognita*	Subventral esophageal glands	Cytoplasm	Disrupts the isochorismate synthase pathway for SA biosynthesis	[108]

## Data Availability

Not applicable.
